# Lanyamycin, a macrolide antibiotic from *Sorangium cellulosum*, strain Soce 481 (Myxobacteria)

**DOI:** 10.3762/bjoc.14.132

**Published:** 2018-06-26

**Authors:** Lucky S Mulwa, Rolf Jansen, Dimas F Praditya, Kathrin I Mohr, Patrick W Okanya, Joachim Wink, Eike Steinmann, Marc Stadler

**Affiliations:** 1 Department of Microbial Drugs, Helmholtz Centre for Infection Research and German Centre for Infection Research (DZIF), partner site Hannover/Braunschweig, Inhoffenstrasse 7, 38124 Braunschweig, Germany; 2 Work group Microbial Strain Collection (MISG), Helmholtz Centre for Infection Research, Inhoffenstrasse 7, 38124 Braunschweig, Germany; 3 TWINCORE-Centre for Experimental and Clinical Infection Research (Institute of Experimental Virology) Hannover. Feodor-Lynen-Str. 7–9, 30625 Hannover, Germany. Tel: +49(0)511-220027-133; 4 Department of Biochemistry and Biotechnology, The Technical University of Kenya, P.O. Box 52428 – 00200, Located along Haile Selassie Avenue, Nairobi, Kenya. Tel: +254(020) 2219929; 5 Department of Molecular and Medical Virology, Ruhr-University Bochum, 44801 Bochum, Germany

**Keywords:** antimicrobial, HCV, lanyamycin, macrolide, *Sorangium cellulosum*

## Abstract

Lanyamycin (**1**/**2**), a secondary metabolite occurring as two epimers, was isolated from the myxobacterium *Sorangium cellulosum*, strain Soce 481. The structures of both epimers were elucidated from HRESIMS and 1D and 2D NMR data and the relative configuration of their macrolactone ring was assigned based on NOE and vicinal ^1^H NMR coupling constants and by calculation of a 3D model. Lanyamycin inhibited HCV infection into mammalian liver cells with an IC_50_ value of 11.8 µM, and exhibited a moderate cytotoxic activity against the mouse fibroblast cell line L929 and the human nasopharyngeal cell line KB3 with IC_50_ values of 3.1 and 1.5 μM, respectively, and also suppressed the growth of the Gram-positive bacterium *Micrococcus luteus*.

## Introduction

Viral hepatitis (HCV) has recently become a major concern of governments and health workers due to the economic and health burden it has brought. HCV has been found to be the main cause of liver associated deaths globally putting it in the same league of HIV and tuberculosis in terms of worldwide health challenges [1]. Approximately 71 million people are infected with Hepatitis C virus. Hepatitis C virus (HCV) is a major cause of liver cirrhosis and hepatocellular carcinoma. Although a variety of potent direct-acting antiviral drugs have been licensed recently, their high costs exclude the majority of infected individuals from treatment. Additionally, the treatment and management of viral and bacterial diseases is complicated by increasing rates of multidrug resistance. Hence, the need for new chemical scaffolds urgently required to increase the chemical diversity of drugs, especially to obtain antibiotics that overcome resistance by new modes of actions [2]. Myxobacteria have emerged as a productive source of antiviral and antimicrobial molecules with unique structures and novel modes of action [3]. The potential of myxobacteria as source of anti-invectives may be illustrated by phenoxan, phenalamide A1, thiangazole and aetheramide A, all of which were found to exert anti-HIV activity while sorazolones, argyrins and tubulysins showed antitumor activity or cytotoxicity [4–8]. In our studies on the secondary metabolites of *Sorangium cellulosum* (strain Soce 481), we observed strong antifungal activity in the raw extract. On RP-HPLC fractionating and peak-activity correlation we further noted at *t*_R_ = 18–25 min an inhibition overlap of *Micrococcus luteus* and *Candida albicans*, which correlated with some prominent peaks with unique HPLC-DAD-MS characteristics attracting our interest. Consequently, the fermentation process was optimized and scaled up. The main active principle was identified as soraphen A, which is already known from *Sorangium cellulosum* [9]. However, on close analysis of peak–activity correlation, we detected another biologically active metabolite inhibiting growth of *Micrococcus luteus* correlating to a distinctive peak and UV spectrum in the HPLC. Its production, isolation, biological and physicochemical characterization is reported in the current paper.

## Results and Discussion

**Figure 1:**
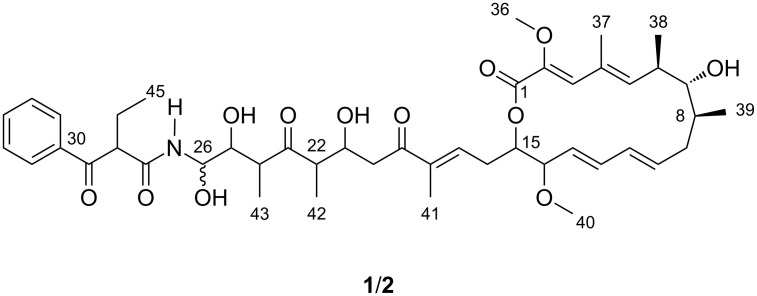
Lanyamycin (**1**/**2**), differing at C-26, isolated from *Sorangium cellulosum* (Soce 481).

Lanyamycin (**1**/**2**, Figure 1) was isolated as a pale-yellow amorphous substance. On silica gel TLC it had an *R*_f_ value of 0.43 with dichloromethane/acetone (3:2) and turned dark brown on treatment with vanillin/H_2_SO_4_ reagent.

**Figure 2:**
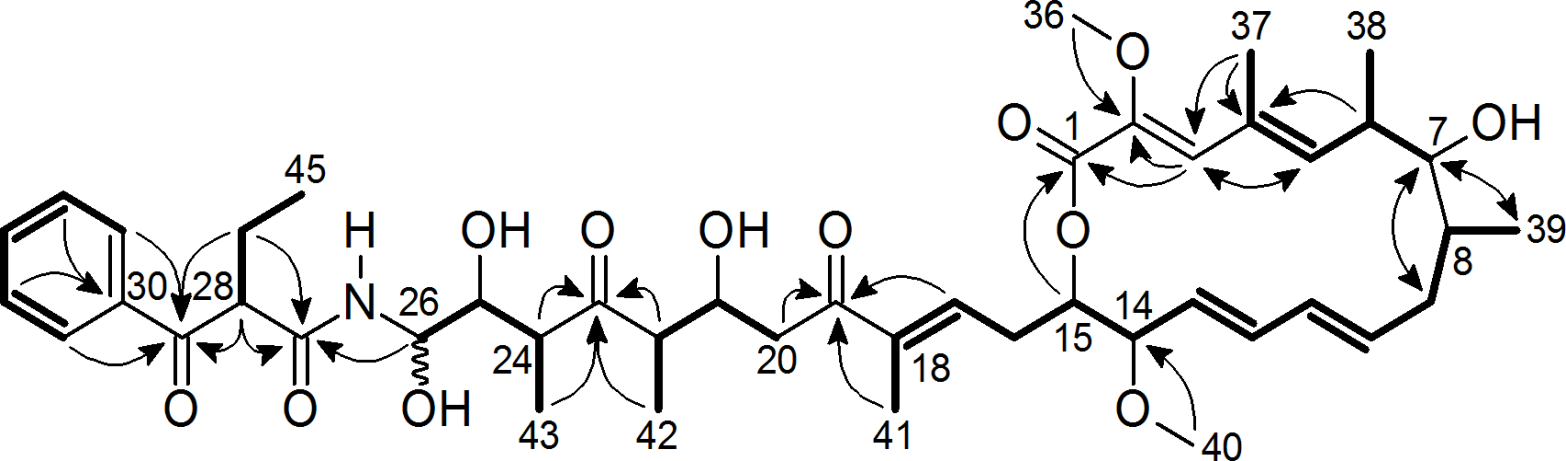
Structure fragments of lanyamycin (**1**/**2**) from ^1^H,^1^H-COSY spectrum (bold bonds) and selected ^1^H,^13^C-HMBC NMR correlations (arrows).

RP-HPLC coupled to HRESIMS showed the two compounds were isomers with the elemental formula C_45_H_63_NO_12_, which was derived from the ion clusters [2M + Na]^+^, [M + Na]^+^, [M − H_2_O + H]^+^, [M − 2H_2_O + H]^+^, and [M − 3H_2_O + H]^+^ with high congruency of measured and calculated ion intensities while the [M + H]^+^ ion cluster was completely absent. In spite of their similarity, the lanyamycin epimers **1** and **2** could be separated by repeated RP-HPLC on Nucleodur Phenyl-Hexyl with an isocratic water/acetonitrile solvent system (52:48). Careful handling of the samples **1** and **2** allowed measuring 1D and 2D NMR data sets in methanol-*d*_4_ of both before a rearrangement finally restored the original mixture. Comparative NMR analyses showed that the spectra of **1** and **2** were very similar (Table 1, and Supporting Information File 1, Tables S1 and S2, Figures S1–S7). 43 signals in the ^13^C NMR spectra supported the elemental formula with 45 carbon atoms, because two aromatic methine signals stood out with double intensity. The signals of 58 protons in the ^1^H NMR spectrum were assigned from ^1^H,^13^C-HSQC-DEPT NMR spectra to their corresponding carbon atoms leaving only 5 exchangeable OH and NH protons. Including long-range correlations, the ^1^H,^1^H-COSY NMR spectra of **1** and **2** provided six main structure fragments (Figure 2). Remarkably, no coupling was observed between methines-7 and -8, even after searching for helpful correlations in the ^1^H,^1^H-TOCSY NMR spectra. This gap was bridged by mutual ^1^H,^13^C-HMBC correlations between methine-7, methylene-9 and methyl-39 (Figure 2, Table 1). Further, the HMBC spectra indicated the positions of two methoxyl groups (C-36, C-40) at δ_C_ 60 and 56 ppm and of four completely substituted sp^2^ carbons (C-2, C-4, C-18, C-30). The closure of the 16-membered lactone was apparent from the HMBC correlation between H-15 and C-1 (δ_C_ 166 ppm) and from the downfield shift of H-15 (δ_H_ 4.81 ppm). Together with four exchanged protons, the oxymethines (C-7, C-21, C-25, C-26) between δ_C_ 72 and 77 ppm were assigned as alcohol positions. The only remaining NH group finally filled the gap between the amide C-27 (δ_C_ 171 ppm) and C-26 (δ_C_ 75 ppm) based on the HMBC correlation H-26/C-27 thus forming the unusual and unstable *N*-acyl hemiaminal that explains the interconversion of lanyamycin isomers **1** and **2**. The *E* configuration of the diene double bonds Δ^10,11^ and Δ^12,13^ was recognized from their large coupling constants of about 15 Hz while ^1^H,^1^H-ROESY correlations between H-3 and H-5 as well as between methyl-37 and both methyl-38 and methoxy-36 suggested the *E* configuration of the substituted Δ^2,3^,Δ^4,5^-diene. Likewise, the *E* configuration of the substituted Δ^17,18^ double bond was derived from the ROESY correlations between methyl-41 and methylene protons 16a and 16b and between methine-17 and methylenes H-20a and H-20b. The latter simultaneously indicated the *s-trans* configuration of the enone and explained the observation of a ROESY correlation between H-17 and H-21.

**Table 1:** NMR data of lanyamycin epimers (**1** and **2**) in CD_3_OD at (150/125 MHz).

		**1**					**2**		

pos.	δ_C_	type	δ_H_	H mult. (*J* [Hz])	pos.	δ_C_	type	δ_H_	H mult. (*J* [Hz])

1	166.06	C			1	166.03	C		
2	143.04	C			2	143.07	C		
3	133.63	CH	6.53	br s	3	133.57	CH	6.55	d (0.7)
4	132.61	C			4	132.62	C		
5	147.62	CH	5.38	d (9.9)	5	147.55	CH	5.39	br d (10.1)
6	39.67	CH	2.39	tq (10.0, 6.6)	6	39.67	CH	2.40	tq (10.0, 6.6)
7	77.22	CH	3.38	br d (10.1)	7	77.24	CH	3.39	br d (10.5)
8	42.99	CH	1.48	m (9.8, 7.0, 3.1)	8	42.99	CH	1.50	dqd (9.9, 6.8, 3.0)
9a	38.78	CH_2_	2.03	m^a^	9a	38.78	CH_2_	2.05	m^a^
9b			1.88	dd (14.5, 6.4)	9b			1.89	br ddd (12.4, 9.9, 2.0)
10	138.29	CH	6.16	ddd (15.3, 9.5, 5.7)	10	138.26	CH	6.17	ddd (15.3, 9.8, 5.7)
11	132.75	CH	6.01	dd (15.1, 10.4)	11	132.78	CH	6.03	dd (15.3, 10.5)
12	139.79	CH	6.50	dd (15.2, 10.6)	12	139.76	CH	6.51	dd (15.2, 10.4)
13	128.95	CH	5.17	dd (15.1, 9.3)	13	128.99	CH	5.20	br dd (15.1, 9.5)
14	85.75	CH	3.76	t (9.5)	14	85.77	CH	3.77	t (9.5)
15	75.84	CH	4.81	ddd (9.8, 6.1, 4.1)	15	75.84	CH	4.83	ddd (10.1, 6.5, 3.7)
16a	33.21	CH_2_	2.86	m^b^	16a	33.20	CH_2_	2.88	dddd (15.7, 5.9, 4.0, 0.9)^b^
16b			2.80	m^c^	16b			2.81	ddd (15.7, 8.8, 6.9)^c^
17	140.25	CH	6.85	br t (7.0)	17	140.16	CH	6.85	ddq (8.6, 6.2, 1.3)
18	140.58	C			18	140.58	C		
19	202.54	C			19	202.53	C		
20a	43.47	CH_2_	2.98	dd (15.5, 2.8)	20a	43.35	CH_2_	2.98	dd (15.5, 3.0)
20b			2.77	dd (15.6, 9.3)^c^	20b			2.78	dd (15.7, 9.3)^c^
21	72.40	CH	4.24	ddd (9.3, 8.0, 2.7)	21	72.10	CH	4.25	ddd (9.2, 8.0, 3.0)
22	51.55	CH	3.07	dq (8.0, 6.9)	22	51.98	CH	3.06	dq (7.8, 7.0)
23	217.46	C=O			23	217.63	C		
24	51.00	CH	2.89	qd (7.0, 5.5)^b^	24	50.73	CH	2.91	qd (6.9, 5.8)^b^
25	74.32	CH	3.89	t (5.5)	25	74.52	CH	3.85	t (5.5)
26	75.08	CH	5.19	br d (5.8)	26	75.03	CH	5.22	d (5.2)
27	171.87	C=O			27	171.66	C		
28	58.25	CH	4.29	dd (7.6, 6.6)	28	58.29	CH	4.34	dd (7.7, 6.7)
29	198.10	C=O			29	198.19	C		
30	138.12	C			30	138.15	C		
33	134.77	CH	7.60	tt (7.4, 1.2)	33	134.81	CH	7.63	tt (7.3, 1.3)
34, 32	130.10	2 CH	7.50	br t (7.8)	32, 34	130.06	CH	7.53	td (7.5, 1.6)
35, 31	129.55	2 CH	7.99	br d (7.5)	31, 35	129.76	CH	8.06	dd (8.4, 1.3)
36	60.79	OCH_3_	3.59	s	36	60.77	CH_3_	3.61	s
37	14.56	CH_3_	1.91	s	37	14.57	CH_3_	1.92	d (1.1)
38	17.05	CH_3_	1.01	br d (6.6)	38	17.06	CH_3_	1.03	br d (6.7)
39	15.57	CH_3_	0.89	d (7.0)	39	15.57	CH_3_	0.90	d (7.1)
40	56.65	OCH_3_	3.27	s	40	56.67	CH_3_	3.29	s
41	11.74	CH_3_	1.79	s	41	11.74	CH_3_	1.81	br s
42	13.84	CH_3_	1.05	d (6.9)	42	13.71	CH_3_	1.06	d (7.1)
43	10.51	CH_3_	0.92	d (7.0)	43	11.28	CH_3_	1.07	d (6.9)
44a	24.28	CH_2_	2.01	m^b,d^	44a	24.62	CH_2_	2.03	m^a^
44b			1.95	m^d^	44b			1.97	m
45	12.55	CH_3_	1.03	t (7.5)	45	12.57	CH3	1.02	t (7.2)

^a, b, c, d^Overlaping ^1^H signals.

For confirmation, NMR spectra in DMSO-*d*_6_ were measured (Supporting Information File 1, Table S3). At the beginning of the NMR experiments isomer **1** was the major component. Later both were present in equal amounts. The ^1^H doublets in the ^1^H NMR spectrum at 8.64 and 8.75 ppm (*J* = 9.5 and 9.3 Hz) were identified as the NH and NH* signals of **1** and **2** from their correlation in the ^15^N,^1^H-HSQC NMR spectrum, respectively. They showed couplings to H-26 (4.96 ppm) and H-26* (4.91 ppm). The latter showed further ^1^H couplings to doublets of OH and OH* signals at 5.89 (5.6 Hz) and 5.95 ppm (5.4 Hz) and thus fully establishing the hemiacetal amide structural part. The mean value of the coupling constants between H-26 and H-25 remained as 5.7 Hz for **1** and 6.4 Hz for **2** indicating both compounds were epimers at C-26. Accordingly, ^13^C and ^1^H shift differences between lanyamycin epimers **1** and **2** mainly were observed in the side chains of the 16 membered macrolactone. As consequence of the epimerization, the conformation of the amide part of the side chain was adjusted. Particularly, the methyl group C-43 next to the epimeric centre was shifted from 0.87 ppm to higher field, i.e., 0.57 ppm, for C-43*. This allowed to observe a strong NOE between C-43 and 25OH, while the NOE between C-43* and 25*OH was only weak. Similarly, only the aromatic 35*/31* proton signals had an NOE with H-24* while the corresponding correlation was absent in isomer **1**. Thus, the high-field shift (Δδ = 0.3 ppm) of C-43* was caused by the anisotropy of the aromatic ring, which requires a reorientation of the amide part of the hemiaminal in isomer **2**.

**Figure 3:**
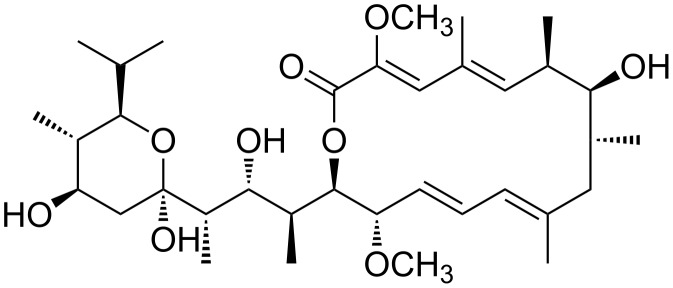
Structure of bafilomycin A_1_.

The macrolactone ring of lanyamycin **1**/**2** was found to be related to those characterizing the bafilomycins (Figure 3) [10–11]. The obvious difference is the missing methyl group of the unsubstituted diene in **1**/**2**. Further comparison of the NMR data revealed a significant lower ^13^C-shift of C-7 of about 5–6 ppm. Together with the missing vicinal coupling between H-7 and H-8 this indicated an inverted configuration of the secondary alcohol at C-7.

**Figure 4:**
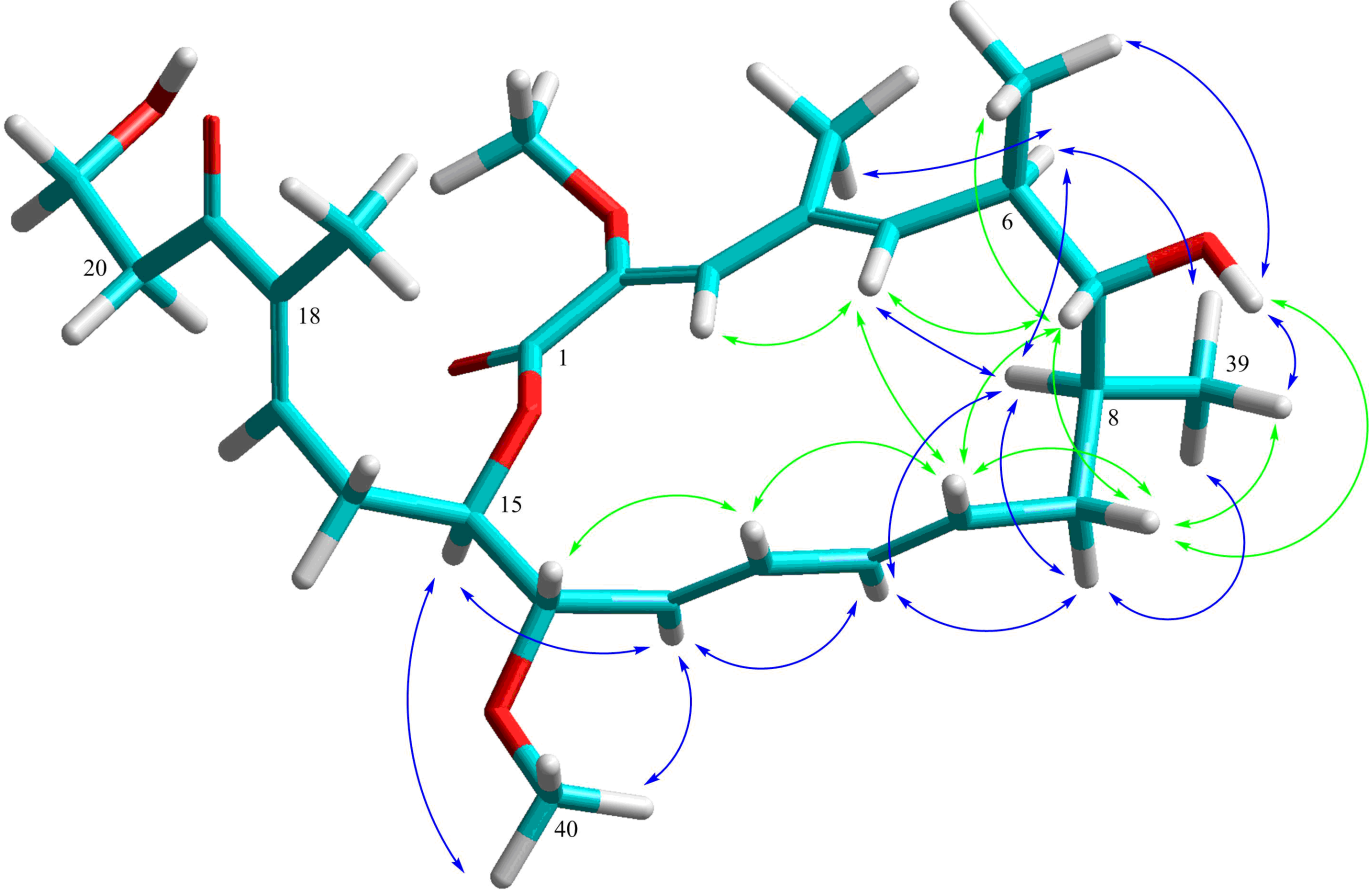
3D Model of the macrolactone ring of lanyamycin (**1**/**2**) and selected ROESY correlations. Green arrows upper side NOEs, blue arrows under side NOEs.

Since the X-ray structure of bafilomycin A_1_ is available from CCDC it was used to calculate the 3D model of the lactone ring in **1**/**2** using HyperChem Prof. (version 8.0.10, Figure 4). Initially the X-ray structure was optimized with the mm+ method, which showed a good congruency of X-ray structure and mm+ model especially of the macrolactone part. After deletion of the methyl group at C-10 and inversion of the C-7 configuration a simplified side chain of **1**/**2** was attached. In the resulting mm+ model of the lactone ring of **1**/**2** the calculated torsion angle between H-7 and H-8 was about 108°, explaining the absence of their vicinal coupling in the 1D and 2D ^1^H NMR spectra. In congruence with the vicinal coupling constants *J*_5/6_ = *J*_6/7_ = 9.9 Hz suggesting the *trans* orientation of their protons, the model provided torsion angles of 161° and 178°, respectively. Similarly the vicinal coupling constants *J*_13/14_ = 9.5 Hz and *J*_14/15_ = 8 Hz indicated the *trans* orientation of their protons in the macrolactone ring with torsion angles of 177° and 170°. Further, the distances between proton pairs showing NOE interactions in the ROESY spectra of **1**/**2** were analyzed (Table 2). These distances showed a very good relation to the ROESY correlations (Supporting Information File 1, Table S3). For example, the series of strong pairwise ROESY correlations of H-3, -5, -7, -10, -12, and H-14 had a maximal distance of 2.4 Å in the model. Thus, the congruency of 3D model and NMR data supported the inversion of the configuration of the secondary alcohol and suggested the relative configuration of the macrolactone ring of lanyamycin (**1**/**2**).

**Table 2:** Atom distances in the 3D model for selected ROESY correlations observed in lanyamycin (**1**/**2**).

atom 1/atom 2	distance [Å]	atom 1/atom 2	distance [Å]

3/5	2.15	8/9b	2.51
5/7	2.42	8/11	2.96
5/8	2.94	9a/10	2.47
5/10	2.68	9a/39	2.69
6/8	2.58	9b/11	2.44
6/37	2.09	9b/39	2.47
6/39	2.77	10/12	2.39
7/7OH	2.15	11/13	2.42
7/9a	2.61	12/14	2.37
7/10	2.39	13/15	2.69
7/38	2.61	13/40	2.92
7-OH/9a	2.82	14/40	2.36
7-OH/38	3.34		
7-OH/39	2.32		

A close analysis of the NOEs revealed some weak long-range correlations suggesting a different folding of epimers **1** and **2** (Supporting Information File 1, Figure S8). In **1** the two rings seem to be bending closer to the centre of the open chain as shown by the NOE correlation pairs of H-36/42, 3/42, 31/43, 35/43, 35/24. These NOEs are not present in the case of **2**.

Aside from their related macrolide rings lanyamycin (**1**/**2**) and bafilomycins, including hygrolidins, micromonospolides and other bafilomycin type antibiotics, show very little similarity in their structures. The typical α-methyl-β-hydroxy part of the bafilomycin side chain is absent in **1**/**2**, which features a unique α-methylene group. However, both share a carbonyl function in δ-position to the macrolide, which often is masked as a hemiketal in bafilomycins, e.g., in bafilomycin A_1_ [11–14].

Bafilomycins are a family of macrolide antibiotics isolated from actinobacteria such as *Micromonospora* and *Streptomyces* species [11]. They are specific inhibitors of vacuolar ATPase (V-ATPase) [15]. The most studied compound of this class, bafilomycin A_1_ (Figure 3) is a useful biochemical tool as it prevents the re-acidification of synaptic vesicles once they have undergone exocytosis [14]. Bafilomycins are also known to be cytotoxic, inhibitors of autophagy, active against Gram-positive bacteria, yeast and fungi, nematodes, insects, immunosuppressant or with antitumor activities [12–15]. Therefore, lanyamycin (**1/2**) was tested in our screening panel against bacteria, fungi, mammalian cell cultures and for antiviral activity against HCV in human liver cells.

Lanyamycin (**1**/**2**) was analyzed for antimicrobial activity against bacteria and fungi and showed moderate antifungal activity in addition to a good inhibition of *Micrococcus luteus* (Table 3).

**Table 3:** Antimicrobial activity of lanyamycin (**1**/**2**).

test strains	MIC [µg/mL]

fungi	

*Candida albicans* (DSM 1665)^a^	4.2
*Mucor hiemalis* (DSM 2656)^a^	4.2
*Rhodotorula glutinis* (DSM 10134)^a^	4.2

Gram-positive bacteria	

*Bacillus subtilis* (DSM 10)^b^	–
*Micrococcus luteus* (DSM 1790)^c^	1.7
*Staphylococcus aureus* (Newman)^d^	–

Gram-negative bacteria	

*Chromobacterium violaceum* (DSM 30191)^b^	–
*Escherichia coli* (DSM 1116)^d^	–
*Escherichia coli* (ToLC)^d^	–

^a^Nystatin, ^b^tetracycline, ^c^ampicillin, ^d^gentamycin.

When lanyamycin (**1**/**2**) was evaluated for cytotoxicity against growing primary cell lines, mouse fibroblasts (L929), and human cancer cell lines, nasopharyngeal cells (KB3.1), colon carcinoma cells (HCT-116), and glioblastoma (U87MG). It exhibited cytotoxic activity with IC_50_ values of 3.1 and 1.5 µM against L929 and KB3.1 cell cultures, respectively, while it was not active against the other cell lines according to the limits of the National Cancer Institute (active IC_50_ values ≤10 µM) [16] (Table 4).

**Table 4:** Cytotoxicity test results of lanyamycin (**1/2**).

cell line	IC_50_ [µM]

mouse fibroblasts (L929)^a^	3.1
human nasopharyngeal cells (KB3.1)^a^	1.5
human colon carcinoma cells (HCT-116)^a^	22
human glioblastoma (U87MG)^a^	29

^a^control: epothilone B.

**Figure 5:**
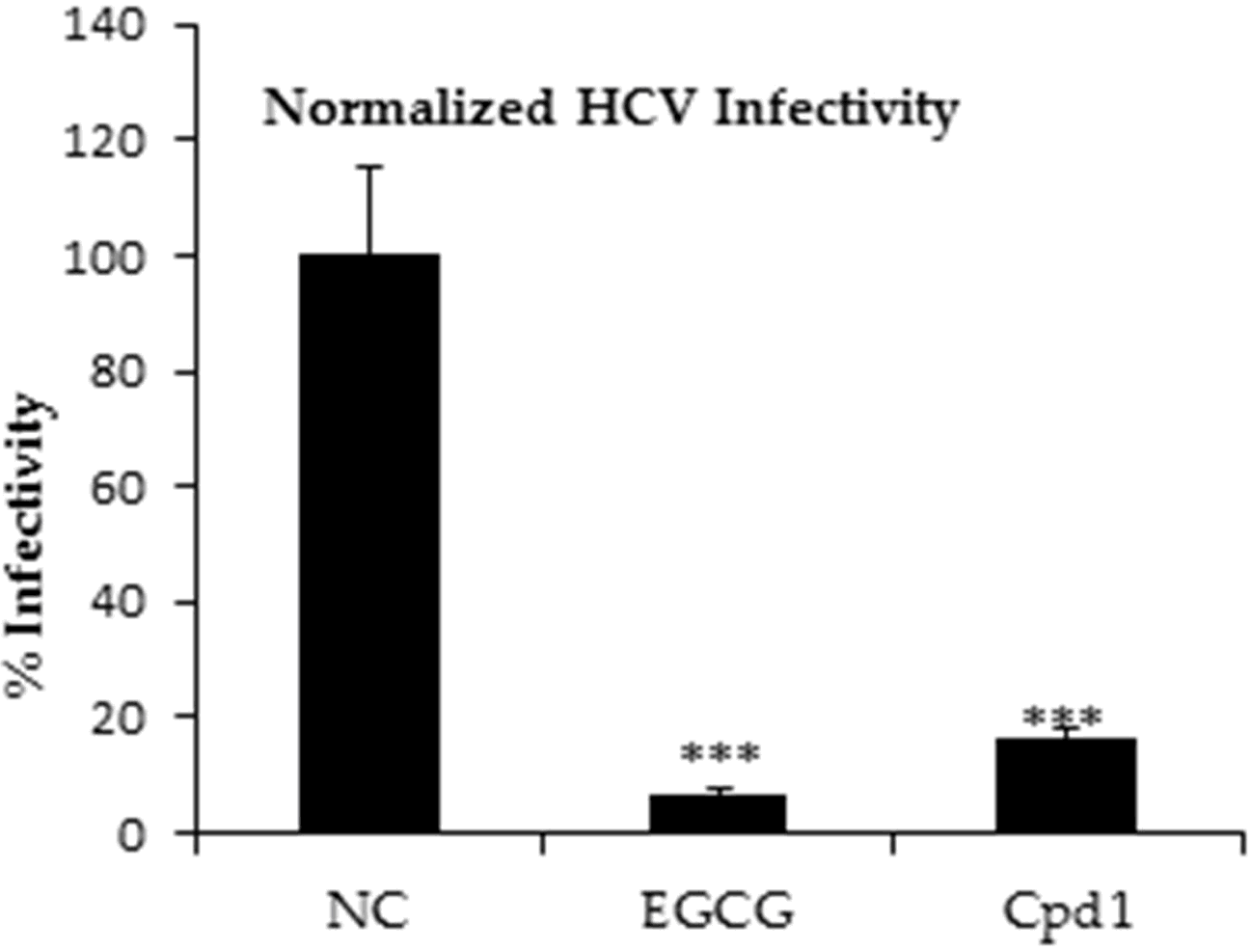
HCV assay: NC = negative control, EGCG = positive control, Cpd1 = lanyamycin (**1**/**2**).

Lanyamycin (**1/2**) showed an inhibition of HCV infection with an IC_50_ value of 11.8 µM, without any cytotoxicity of the human liver cells, which was simultaneously determined (see Figure 5 and for viability assay, Supporting Information File 1, Figures S9 and S10). The green tea molecule epigallocatechin gallate (EGCG) was used as positive control (IC_50_ value of 5–21 µM) [17], as previously described [18].

## Conclusion

The new compound lanyamycin (**1**/**2**) showed significant, but rather weak antiviral effects, as well as moderate cytotoxic activities, which were, however, not noted against the cell line that was used in the antiviral assay. The activity profile is different from that of the bafiloymcins. Interestingly, the present report is the first discovery of a bafilomycin-like molecule from an organism that does not belong to the Actinobacteria. Follow-up studies on its biosynthesis and comparison of the homology of the respective gene clusters appear interesting in order to address the hypothesis that a horizontal gene transfer of the biosynthesis gene cluster between an actinobacterium and a myxobacterium may have occurred.

## Experimental

### General experimental procedures

Analytical TLC: TLC aluminum sheets silica gel Si 60 F_254_ (Merck), detection by UV absorption at 254 nm and 366 nm. Spray reagent vanillin/sulfuric acid (prepared by dissolving vanillin (0.5 g) in 100 mL of sulfuric acid) followed by heating to about 120 °C on a hot plate. UV data were recorded on a Shimadzu UV/vis-2450 spectrophotometer using methanol (UVASOL, Merck) as solvent. Optical rotation was measured using a Perkin–Elmer 241 polarimeter. NMR spectra were recorded on a Bruker Avance DMX 600 or DPX 500/700 NMR spectrometer, locked to the deuterated signal of the solvent. Data acquisition, processing, and spectral analysis were performed with standard Bruker software and ACD/NMR workbook. Chemical shifts are given in parts per million (ppm), and coupling constants in hertz (Hz). HRESIMS data were recorded on a Maxis ESI TOF mass spectrometer (Bruker Daltonics); molecular formulas were calculated including the isotopic pattern (Smart Formula algorithm). Analytical RP HPLC was carried out with an Agilent 1260 system equipped with UV diode-array detector and Corona Ultra detector (Dionex). HPLC conditions: column 125 × 2 mm, Nucleodur C18, 5 μm (Macherey-Nagel), solvent A: 5% acetonitrile in water, 5 mmol NH_4_Ac, 0.04 mL/L AcOH; solvent B: 95% acetonitrile, 5 mmol NH_4_Ac, 0.04 mL/L AcOH; gradient system: 10% B increasing to 100% B in 30 min, 10% B for 10 min, to 10% B post-run for 10 min; 40 °C; flow rate 0.3 mL/min.

### Cultivation of *Sorangium cellulosum* Soce 481

*Sorangium cellulosum*, strain Soce 481, was isolated in 1990 from a soil sample with plant residue collected at Alanya in Turkey in 1986 and stored at −80 °C. It was reactivated in 20 mL of liquid medium composed of 0.1% soya meal, 0.15% casitone, 0.1% yeast extracts, 0.1% CaCl_2_, 50 mM HEPES (11.9 g), 0.1% glucose, 0.4% starch, 0.5% MgSO_4_ and 4 mg/L Fe-EDTA, pH 7.4. The culture was upscaled to 1 L and used to inoculate 40 L of production media in 2 L shake flasks with 1 L of medium and 2% Amberlite XAD 16 absorber resin (Rohm and Haas, Frankfurt, Germany) [19]. The production medium was composed of 0.4% soya meal, 0.2% glucose, 0.8% starch, 0.1% CaCl_2_, 0.1% MgSO_4_, 11.9% yeast extract, 1 mL/L Fe-EDTA, pH 7.4. Each flask was inoculated with 100 mL of well-grown 5-day culture, and then incubated in shake flask on a rotary shaker (160 rpm) at 25 °C for 14 days as recently described in [18].

### Isolation of compounds **1** and **2**

The fermentation was stopped on the 15th day. The Amberlite XAD 16 resin was collected by sieving, washed by flushing with distilled water, and then extracted in a glass column (70 × 8 cm) with methanol (ca. 6 L) followed by acetone (2 L) at a flow rate of 30 mL/min. The extract was evaporated in a rotary evaporator giving 15.4 g of an oily crude extract. Enrichment of the target compounds was achieved by eliminating the highly polar and the lipophilic compounds by consecutive partitioning with ethyl acetate/water. The ethyl acetate phase was dried and partitioned further in methanol/*n*-heptane ending up with 2.9 g of enriched methanol extract. This extract was purified further by Si-Flash chromatography (Grace Reveleris^®^) using an 80 g silica column, with dichloromethane (DCM)/acetone solvent gradient of 25% to 100% acetone in 23 min followed by 100% acetone for 13 min. This resulted in 9 fractions collected according to their UV profile. Fraction 5 (219 mg) indicated the presence of compound **1/2** among other compounds by HPLC analysis. Fraction 5 was further purified by preparative RP-HPLC, using a Nucleodur Phenyl-Hexyl, 5 µm column, 250 × 21 mm (Macherey-Nagel, Düren, Germany), water and acetonitrile solvent system (isocratic) at 48% acetonitrile and 52% water for 1 hour with a flow rate of 20 mL/min and UV detection at 254 nm. Compound **1** (5.6 mg) eluted with a retention time of *t_R_* = 20.8 min and its epimer, **2** (2.8 mg) at *t_R_* = 22.3 min. Both peaks overlapped slightly at the centre giving 15.2 mg of a mixture of epimers **1** and **2**.

**Lanyamycin** (**1**/**2**): C_45_H_63_NO_12_ M = 809 g/mol: [α]_D_^22^ = +44° and [α]_D_^22^ = −136° (*c* 0.5g/L, methanol), respectively; UV (methanol) λ_max_ (log ε): 282 (4.407), 243 (4.821), 268 (4.335) nm; analytical RP-HPLC two peaks at 12.68 and 12.75 min with identical HRESIMS data: [2M + Na]^+^ calcd for C_90_H_126_N_2_NaO_24_, 1641.8592 (mσ 7.4); found, 1641.8607; [M + Na]^+^ calcd for C_45_H_63_NNaO_12_, 832.4242 (mσ 3.6); found, 832.4255 [M − H_2_O + H]^+^ calcd for C_45_H_62_NNaO_11_, 792.4317 (mσ 2.1); found, 792.4329; [M − 2H_2_O + H]^+^ calcd. for C_45_H_60_NNaO_10_, 774.4212 (mσ 117 due to overlap); found, 774.4228; [M − 3H_2_O + H]^+^ calcd for C_45_H_58_NNaO_9_, 756.4106 (mσ 8.8); found, 756.4120. NMR data in Table 1, Supporting Information File 1, Tables S1–S3 and Figure S1–S7.

### Inhibitory effects on HCV infectivity

Huh-7.5 cells were inoculated with RLuc Jc1 reporter viruses in the presence of different compounds. The inoculum was removed 4 hours later and monolayers were washed three times with PBS and overlaid with fresh medium containing no inhibitors. Infected cells were lysed 3 days later, and reporter virus infection was determined by renilla luciferase activity. The cell viability was measured by determination of firefly luciferase. The assay was performed in quadruplicate and is presented as the mean ± standard deviation. *** *P* ≤ 0.05. Viability assay results are given in Supporting Information File 1, Figure S9. Huh-7.5 cells stably expressing Firefly luciferase (Huh-7.5 Fluc) were cultured in Dulbecco’s modified minimum essential medium (DMEM, Life Technologies Manchester UK) containing 2 mM/L glutamine, 1 × minimum essential medium nonessential amino acids (MEM NEAA, Life Technologies), 100 μg/mL streptomycin, 100 IU/mL penicillin (Life Technologies), 5 μg/mL blasticidin and 10% fetal bovine serum. Cells were maintained in a 37 °C environment with 5% CO_2_ supply. Cells were infected with Jc1-derived Renilla reporter viruses in the presence or absence of compounds. Infected cells were lysed and then frozen at −80 °C for 1 hour following measurements of Renilla and Firefly luciferase activities on a Berthold Technologies Centro XS3 Microplate Luminometer as indicators of viral genome replication and cell viability, respectively, as previously described [18].

### Cytotoxic activity

The cytotoxicity was evaluated by measuring the effect produced on cell morphology including the nuclei and cell growth in vitro. Cell monolayers were prepared in 96-well microtiter plates and exposed to various concentrations of the compounds. The experiment was done in duplicate. Plates were checked by light microscopy after 24, 48 and 72 hours and on the 5th day, then cytotoxicity was scored as morphological alterations (e.g., rounding up, shrinking, detachment and disintegration of nuclei). The viability of the cells was determined by a tetrazolium-based colorimetric method using 3-(4,5-dimethylthiazol-2-yl)-2,5-diphenyltetrazolium bromide (MTT), as previously described [18]. The 50% cytotoxic dose, IC_50_, is the concentration of the compound that reduced the absorbance of the control sample by 50%. The cell culture was diluted to 50,000 cells per well. 120 µL of cell suspension was dispensed in every well in cell culture plate. Then a dilution plate was prepared by adding 100 µL media and 50 µL of test compound (1 mg/mL) in the first column of the plate. 100 µL medium was added into second to twelfth column wells. A serial dilution was done by transferring 50 µL from column 1 to column 2 cells and mixing twice, this was repeated all the way to the 12th column wells. Methanol was used as a negative control and epothilone-B as a positive control. The experiment was done in duplicate. 60 µL from the dilution plate was transferred to the respective well of the cell culture plate ending up with 180 µL of cell suspension plus diluted compound. The cell plate was incubated for 5 days at 37 °C and evaluated under a microscope for cell and nuclei morphological alterations regularly. 20 µL of MTT solution (5 mg/mL) was added to every well in the cell culture plate and incubated at 37 °C for 2–4 hours. The plate was centrifuged at 3000 rpm for 5 min, then 50–100 µL of PBS was added to each well. The plate was centrifuged again then the supernatant was discarded. 50–100 µL of isopropanol with 40 mM HCL was added to solubilize MTT crystals then the plate was shaken at 450–700 rpm on a plate shaker. The absorbance was measured at 595 nm to determine dead and living cells.

### Antimicrobial testing

EBS or MYC media were diluted and standardized with microbial suspensions to about 1.0 × 10^6^ to 2.0 × 10^6^ cells of test organism per mL measured using a calibrated photometer. The MIC values were determined in 96-well microtiter plates by 1:1 serial dilution in EBS medium (0.5% casein peptone, 0.5% protease peptone, 0.1% meet extract, 0.1% yeast extract, pH 7.0) for bacteria and MYC medium (1.0% glucose, 1.0% phytone peptones, 50 mM HEPES (11.9 g/L), pH 7.0) for fungi as previously described [20]. 150 µL of the inoculated medium was dispensed in each well in 96-well microtiter plate. 20 µL and 2 µL aliquots of 1 mg/mL of test compound or control drug was added on the first row wells then diluted by adding 150 µL of fresh medium, mixed, then 150 µL withdrawn and transferred to the next row wells. This was repeated sequentially up to the lowest well. Then 150 µL was discarded from the last well giving a dilution gradient of 67, 33.3, 16.6, 8.3, 4.2, 2.1, 1.0, and 0.52 µg/mL for the 20 µL aliquot and 6.7, 3.3, 1.7, 0.83, 0.42, 0.21, 0.1, 0.052 µg/mL for the 2 µL aliquot. All plates were wrapped with parafilm and placed on a microplate shaker (SB: Heidolph Titramax 1000) incubated for 16 hours at 800 rpm, at 30 °C [20].

## Supporting Information

File 1 Tables of NMR data and figures of the ^1^H and ^13^C NMR including the HCV infectivity results showing viability.
