# Supplementary material for: Lanyamycin, a macrolide antibiotic from Sorangium cellulosum, strain Soce 481 (Myxobacteria)
Source: Beilstein J Org Chem. 2018 Jun 26;14:1554–62. doi: 10.3762/bjoc.14.132 (PMC6037015; doi:10.3762/bjoc.14.132)
Supplement: File 1 — Tables of NMR data and figures of the 1H and 13C NMR including the HCV infectivity results showing viability. [file Beilstein_J_Org_Chem-14-1554-s001.pdf]

Supporting Information

for

Lanyamycin, a macrolide antibiotic from *Sorangium cellulosum*, strain Soce 481 (Myxobacteria)

Lucky S. Mulwa[1,2], Rolf Jansen[1], Dimas F. Praditya[3], Kathrin I. Mohr[1,2], Patrick W. Okanya[4], Joachim Wink[2], Eike Steinmann[3,5] and Marc Stadler [1,*,§]

Address: [1]Department of Microbial Drugs, Helmholtz Centre for Infection Research and German Centre for Infection Research (DZIF), partner site Hannover/Braunschweig, Inhoffenstrasse 7, 38124 Braunschweig, Germany, [2]Work group Microbial Strain Collection (MISG), Helmholtz Centre for Infection Research, Inhoffenstrasse 7, 38124 Braunschweig, Germany, [3]TWINCORE-Centre for Experimental and Clinical Infection Research (Institute of Experimental Virology) Hanover. Feodor-Lynen-Str. 7–9, 30625 Hannover, Germany. Tel: +49(0)511-220027-133, [4]Department of Biochemistry and Biotechnology, The Technical University of Kenya, P.O. Box 52428 – 00200, Located along Haile Selassie Avenue, Nairobi, Kenya. Tel: +254(020) 2219929 and [5]Department of Molecular and Medical Virology, Ruhr-University Bochum, 44801 Bochum, Germany

[*] Corresponding author

Email: Marc Stadler - marc.stadler@helmholtz-hzi.de

[§]Tel.: +49 531 6181-4240; Fax: +49 531 6181 9499

Tables of NMR data and figures of the ^{1}H and ^{13}C NMR including the HCV infectivity results showing viability

Table S1. NMR data of lanyamycin **1** in CD_3OD (^1H/^{13}C NMR at 500.3/125.8 MHz)

Pos.	δ_C	type	δ_H	H Mult. (*J* [Hz])	H in COSY	H in N/ROESY	H to C HMBC
1	166.06	C=O					15, 3
2	143.04	C					36, 3
3	133.63	CH	6.53	br s	5	5 >>37, 42	37, 5
4	132.61	C					6
5	147.62	CH	5.38	d (9.9)	37, 6, 3	3 >7 >38, 8 >10	38, 37, 6, 7, 3
6	39.67	CH	2.39	tq (10.0, 6.6)	38, 7, 5	37, (38) >8, 39	38
7	77.22	CH	3.38	br d (10.1)	6	5, 10 >9a, (8), 38 >11	39, 38, 9, 9, 6, 5
8	42.99	CH	1.48	m (9.8, 7.0, 3.1)	39, 9a, 9b	(39) >5, 6, (7) >11	39, 9, 9, 7
9a	38.78	CH_2	2.03	m[c]	8, 9b, 10	39, 38, 9, 7, 11, 10	39, 7, 11
9b			1.88	dd (14.5, 6.4)	8, 9a, 10	39, 8, 9, 11, 10	39, 7, 11
10	138.29	CH	6.16	ddd (15.3, 9.5, 5.7	9a, 9b, 11	8, 9, 9, 7, 5, 12	11, 12
11	132.75	CH	6.01	dd (15.1, 10.4)	10, 12	8, 9, 9, 7, 13	13, 10
12	139.79	CH	6.50	dd (15.2, 10.6)	13, 11	40, 14, 10	14, 11, 10
13	128.95	CH	5.17	dd (15.1, 9.3)	14, 12	40, 14, 11	11
14	85.75	CH	3.76	t (9.5)	15, 13	41, 16, 16, 40, 15, 13, 12, 17	40, 12
15	75.84	CH	4.81	ddd (9.8, 6.1, 4.1)	16a, 16b, 14	16, 16, 14, 17	16, 14
16a	33.21	CH_2	2.86	m[a]	41, 16b, 15, 17	41, 14, 15, 17	14
16b			2.80	m[b]	41, 16a, 15, 17	41, 14, 15	14
17	140.25	CH	6.85	br t (7.0)	41, 16a, 16b	42, 20, 16, 20, 14, 21, 15	41, 16
18	140.58	C					41, 20
19	202.54	C=O					41, 20, 20, 17
20a	43.47	CH_2	2.98	dd (15.5, 2.8)	20, 21	42, 20, 21, 17	22
20 b			2.77	dd (15.6, 9.3)	20, 21	42, 24, 20, 17	22
21	72.40	CH	4.24	ddd (9.3, 8.0, 2.7)	20a, 20b, 22	42, 20, 17	42, 20, 22
22	51.55	CH	3.07	dq (8.0, 6.9)[b]	42, 21	43, 42, 24, 25	42, 20
23	217.46	C=O					43, 42, 24, 22
24	51.00	CH	2.89	qd (7.0, 5.5)[a]	43, 25	43, 42, 20, 22, 25, 26, 35, 31	43, 25
25	74.32	CH	3.89	t (5.5)	24, 26	43, 42, 24, 22, 26	43, 24
26	75.08	CH	5.19	br d (5.8)	25	43, 42, 24, 25	25
27	171.87	C=O					44, 28, 26
28	58.25	CH	4.29	dd (7.6, 6.6)	44a, 44b	45, 35, 31	45, 44, 44
29	198.10	C=O					44, 44, 28, 35, 31
30	138.12	C					34, 32
33	134.77	CH	7.60	tt (7.4, 1.2)	34, 32, 35, 31		35, 31
34, 32	130.10	2 CH	7.50	br t (7.8)	33, 35, 31	35, 31	
35, 31	129.55	2 CH	7.99	br d (7.5)	34, 32, 33	24, 28, 34, 32	33
36	60.79	OCH_3	3.59	s		42, 37	
37	14.56	CH_3	1.91	s	5	38, 6, 36, 3	5, 3
38	17.05	CH_3	1.01	br d (6.6)	6	37, 9, 6, 7, 5	37, 6, 7
39	15.57	CH_3	0.89	d (7.0)	8	8, 9, 9, 6, 7	7
40	56.65	OCH_3	3.27	s		41, 14, 13, 12	14
41	11.74	CH_3	1.79	s	16a, 16b, 17	16, 16, 40, 14	17
42	13.84	CH_3	1.05	d (6.9)	22	20, 24, 20, 22, 36, 25, 21, 26, 3, 17	22
43	10.512	CH_3	0.92	d (7.0)	24	24, 22, 25, 26	24, 25
44a	24.28	CH_2	2.01	m[c, d]	45, 28		45, 28
44b			1.95	m[d]	45		45, 28
45	12.55	CH_3	1.03	t (7.5)	44a, 44b	28	44, 44, 28

[a, b, c, d] overlapping ^1H signals

Table S2. NMR data of lanyamycin **2** in CD_3OD ($^{1}H/^{13}C$ NMR at 700.44/176.14 MHz)

Pos.	δ_C	XHn	δ_H	H Mult. (*J* [Hz])	H in COSY	H in N/ROESY	H to C HMBC
1	166.03	C					3
2	143.07	C					36, 3
3	133.57	CH	6.55	d (0.7)	-	5	37, 5
4	132.62	C					37
5	147.55	CH	5.39	br d (10.1)	6 >37	3, 7 >10, 38, 6, 8	38, 37, 6, 7, 3
6	39.67	CH	2.40	tq (10.0, 6.6)	38, 7, 5	37 >39, 5, 8	38, 5
7	77.24	CH	3.39	br d (10.5)	6	5, 10 >9a, 8, 38, 6	39, 38, 9, 9, 6, 5
8	42.99	CH	1.50	dqd (9.9, 6.8, 3.0)	39, 9a	39, 7 >6, 10, 11 >5	39, 9, 9, 7
9a	38.78	CH_2	2.05	m[c]	8, 9b, 10	39 >7, 10, 11	39, 8, 7, 11, 10
9b			1.89	ddd (12.4, 9.9, 2.0)	9a, 10	39 >11, 10	39, 8, 7, 11, 10
10	138.26	CH	6.17	ddd (15.3, 9.8, 5.7)	9b, 11 >9a	7, 12 >5 >8, 9a	9, 9, 12
11	132.78	CH	6.03	dd (15.3, 10.5)	10, 12	13 >9b	9, 9, 13, 12
12	139.76	CH	6.51	dd (15.2, 10.4)	13, 11	14, 10 >40	14, 11, 10
13	128.99	CH	5.20	br dd (15.1, 9.5)	12 >14	11 >14, 40	11
14	85.77	CH	3.77	t (9.5)	15, 13	40 >12, 16a, 16b, 17	16, 40, 15, 12
15	75.84	CH	4.83	ddd (10.1, 6.5, 3.7)	14 >16a, 16b	16a, 16b, 14, 17	16, 16, 14, 13, 17
16a	33.20	CH_2	2.88	m[a]	16, 15, 17	15, 41, 14, 17	14, 17
16b			2.81	ddd (15.7, 8.8, 6.9)[b]	16, 15, 17	41, 14, 17, 15	14, 17
17	140.16	CH	6.85	ddq (8.6, 6.2, 1.3)	16a, 16b >41	20a, 20b >15, 21, 14, 16a	16
18	140.58	C					41, 16
19	202.53	C					41, 20, 20, 21, 17
20a	43.35	CH_2	2.98	dd (15.5, 3.0)	20b, 21	20b, 21, 17 >42	22
20b			2.78	dd (15.7, 9.3)[b]	20a, 21	20a, 24 >17 >21	22
21	72.10	CH	4.25	ddd (9.2, 8.0, 3.0)	20a, 20b, 22	42, 20a, 24, 22, 17	42, 20, 20, 22
22	51.98	CH	3.06	dq (7.8, 7.0)	42 >21	42 >24, 25, 21	42, 20
23	217.63	C					42, 24, 22, 25, 21
24	50.73	CH	2.91	qd (6.9, 5.8)[a]	43, 25	42 >22, 25 >26	25
25	74.52	CH	3.85	t (5.5)	24, 26	43, 24, 22, 26	43, 24, 26
26	75.03	CH	5.22	d (5.2)	25	43, 25 >24	25
27	171.66	C					44, 44, 28, 26
28	58.29	CH	4.34	dd (7.7, 6.7)	44, 44	31/35, 44a, 44b >45	45, 44, 44
29	198.19	C					44, 44, 28, 31, 35
30	138.15	C					32, 34
31, 35	129.76	CH	8.06	dd (8.4, 1.3)	32/34, 33	32/34 >28	33
32, 34	130.06	CH	7.53	td (7.5, 1.6)	33, 31/35	31/35, 33	
33	134.81	CH	7.63	tt (7.3, 1.3)	32/34 >31/35	32/34	32, 34, 31, 35
36	60.77	CH_3	3.61	s	-	37	3
37	14.57	CH_3	1.92	d (1.1)	5	38, 6, 3	5, 3
38	17.06	CH_3	1.03	br d (6.7)	6	37, 7, 5	37, 6, 7, 5
39	15.57	CH_3	0.90	d (7.1)	8	8 >9a, 9b, 6	8, 7
40	56.67	CH_3	3.29	s	-	14 >13, 12, 41	14
41	11.74	CH_3	1.81	br s	17	16a, 16b, 40	17
42	13.71	CH_3	1.06	d (7.1)	22	24, 20', 22, 21	22, 21
43	11.28	CH_3	1.07	d (6.9)	24	25, 26	24, 25
44a	24.62	CH_2	2.03	m[c]	45, 44b, 28	28	45, 28
44b			1.97	s	45, 44b, 28	28	45, 28
45	12.57	CH3	1.02	t (7.2)	44, 44	28	44, 44, 28
			4.59	s			

[a, b, c, d] overlapping ^{1}H signals

Table S3. NMR data of lanyamycin **1**/**2** in DMSO-d_6. ($^{1}H/^{13}C/^{15}N$ = 700/176/70.99 MHz)

Pos.	$\delta_{C(N)}$	m	δ_H	m (*J* [Hz])	ROESY[b]	H to C HMBC
1	163.34	C				3 >15
1*	163.36	C				3 >15
2	141.12	C				36, 3 >37
3	130.99	CH	6.39	s	5 >	37, 5
3*	130.96	CH	6.40	s	5 >	37, 5
4	130.07	C				37 >6
5	146.54	CH	5.37	br d (9.9)	3 >7 >38, 8, 10	38, 37, 3 >6, 7
6	38.04	CH	2.23	tq (9.9, 6.7)	38, 37 >39, 8 >7OH	38, 7, 5, 7OH
7	74.31	CH	3.25	2x dd (9.9, 6.2)	7OH >5, 10, 38 >9a	39, 38, 9a >5 >7OH
7OH		OH	4.49	2x d (6.7)	7 >39, 38 >6	-
8	41.35	CH	1.34	m	39 >9b, 6 >5, 11	39, 9a, 9b, 7OH
9a	37.28	CH_2	1.95	m	9b, 39, 10 >7, 7OH	39, 7, 11 >10
9b		CH_2	1.79	M[f]	9a, 11, 39, 8	
10	136.93	CH	6.13	br ddt (15.3, 9.7, 5.9)	12, 9a >5	11, 12 >9a, 9b
11	130.93	CH	6.01	dd (15.3, 10.5)	9b, 13 >8	13 >9a, 9b
12	137.74	CH	6.48	dd (15.3, 10.5)	14, 10	14 >10
13	127.47	CH	5.18	dd (15.3, 9.5)	11, 15 >40	11
14*	83.59	CH	3.72	dd (9.3, 8.0)	40, 12 >15, 16b	40, 12 >16*b
14	83.54	CH	3.74	dd (9.3, 8.0)	40, 12 >15, 16b	40, 12 >16b
15*	73.69	CH	4.71	m	16*a, 16*b, 13 >17*	14, 16b >13, 17, 17*
15	73.67	CH	4.71	m	16a, 16b, 13 >17	
16a, 16*b	31.69	CH_2	2.77	m[d]	overlap 40a/40b	14, 17, 17*
16b, 16*b		CH_2	2.69	m[w]	overlap 40a/40b	-
17	137.48	CH	6.69	ddq (8.2, 6.8, 1.2)	20a, 20b, 15 >21	41 >20
17*	137.55	CH	6.72	ddq (8.0, 6.8, 1.2)	20*a, 20*b, 15/15* >21*	41* >20*
18/18*	138.59	C, C			-	41, 41*, 16b; 16*b
19	199.87	C			-	41 >17 >20b
19*	199.91	C			-	41* >17* >20*b
20a	42.22	CH_2	2.78	m[d]	overl. with 16a/16b, 24	22, 21OH
20b		CH_2	2.69	m	overl. with 16a/16b, 24	22, 21OH
20*a	42.40	CH_2	2.79	m	overl. with 16a/16b, 24	22*, 21*OH
20*b		CH_2	2.73	m[e]	overl. with 16a/16b, 24	22*, 21*OH
21	69.78	CH	4.10	dddd (8.8, 8.0, 5.8, 3.3)	21OH, 22, 20b >20a	42 >20b, 22 >21OH
21*	70.14	CH	4.13	dddd (8.4, 8.0, 6.0, 3.4)	21*OH, 22*, 20*a	42* >20*, 22* >21*OH
21OH		OH	4.77	d (5.6)	21 >22	-
21*OH		OH	4.88	d (6.0)	21*	-
22	49.91	CH	2.99	quin (7.0)	42, 25 >21, 21OH	42 >21OH >20b
22*	49.20[1]	CH	3.06	quin (7.0)	42*, 25* >21*, 21*OH	42* >21*OH, 20*b
23	214.61	C			-	43, 42 >22 >24
23*	214.29	C			-	43* >42*, 22*
24	49.20[1]	CH	2.77	m[d]	overl. with 24*, 20a, 16a and 20a*, 16*a	43, 25OH
24*	49.58	CH	2.78	m[d]	overl. with 24, 20a, 16a and 20*a, 16 a *	43*, 25*OH
25	72.55	CH	3.70	br q (5.6)	25OH, 22, 24 >43, 28, 26OH, NH	43, 26OH >25OH
25*	72.12	CH	3.83	br dt (4.4, 6.2)	25*OH, 22*, 24* >26*, 26*OH, NH*	43*, 26*OH >25*OH,
25OH		OH	4.78	d (6.0)	25, 43	-
25*OH		OH	4.72	d (5.6)	25* >43*	-
26	73.15	CH	4.96	dt (9.1, 5.8)	26OH, 43 >25 >>NH	26OH
26*	73.28	CH	4.91	ddd (9.2, 6.7, 5.4)	26*OH, 43* >25* >>NH*	26*OH
26OH		OH	5.89	d (5.6)	26	-
26*OH		OH	5.95	d (5.4)	26*	-
NH	(137.82)	NH	8.64	d (9.3)	28 >31/35, 25 >24, 26	-
NH*	(138.56)	NH	8.75	d (9.5)	28* >31*/35*, 25* >24*, 26*	-
27	168.33	C			-	28, NH, 44a >26*
27*	168.60	C			-	28*, NH*, 44*a >26*
28	56.01	CH	4.32	br t (7.0)	31/35, NH >45	45 >44a, 44b
28*	56.14	CH	4.27	dd (7.3, 6.7)	31*/35*, NH* >45	45* >44*b, 44*b
29	195.51	C			-	35/31, 28, >44b
29*	195.48	C			-	35*/31*, 28* >44*b
30	136.45	C			-	34/32
30*	136.26	C			-	34*/32*
33	133.17	CH	7.63	m		34/32, 35/31

33*	133.08	CH	7.55	m		34*/32*, 35*/31*
34/32	128.68	CH	7.51	m	35/31	34/32
34*/32*	128.68	CH	7.47	m	35*/31*	34*/32*
35/31	128.20	CH	8.00	m	32/34, 28	33, 35/31
35*/31*	127.87	CH	7.94	m	32*/34*, 28* >24*	33*, 35*/31*
36	59.41	CH_3	3.51	2x s	37	>>3
37	13.91	CH_3	1.82	br s		5, 3
38	16.56	CH_3	0.93	d (6.5)[g]	6, 37, >7OH >7, 5	7
39	15.30	CH_3	0.79	d (7.1	8, 7OH >9b >6,	7
40	55.61	CH_3	3.19	2x s	14 >13 >12	14
41	11.32	CH_3	1.69	s	16a/16b, 40	17
41*	11.35	CH_3	1.71	s	16*a/16*b, 40	17*
42	12.49	CH_3	0.88	d (6.9) [ac h]	22, 22' overl. 24	22
42*	12.68	CH_3	0.89	d (6.9) [a,c t]	22*, 20*a overl. 24*	22*
43	9.83	CH_3	0.87	d (6.9) [a h]	25OH, 26, 24	24, 25
43*	8.59	CH_3	0.57	d (6.9) [a]	26*, 24* >25*OH	24*, 25*
44*a	21,80	CH_2	1.88	m	45*, 28*	45*, 28*
44*b		CH_2	1.77	M[f]	45*, 28*	
44a	22.29	CH_2	1.85	M[f]	45, 28	45, 28
44b		CH_2	1.78	m	45, 28	
45	11.79	CH_3	0.88	t (7.4) [a h]	28	28, 44
45*	11.83	CH_3	0.94	t (7.3) [a g]	28*	28*, 44*

[a] from *J*res-Spectrum; [b] sorted with decreasing intensity; [c] interchangeable ^{1}H; [d, e, f, g, h] overlapping ^{1}H signals

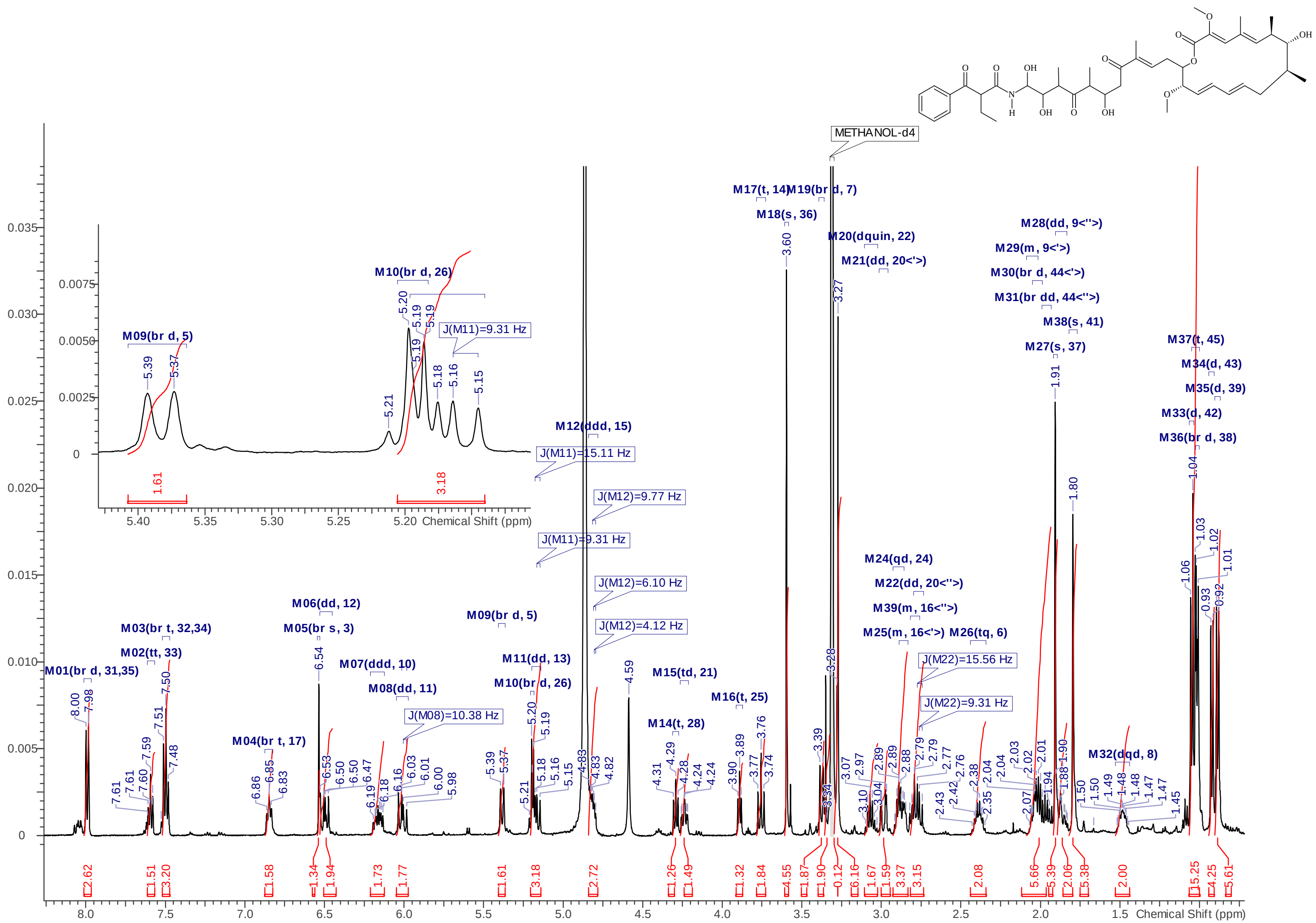

Figure S1. ^{1}H NMR spectrum of lanyamycin (**1**) in CD_3OD (500.3 MHz)

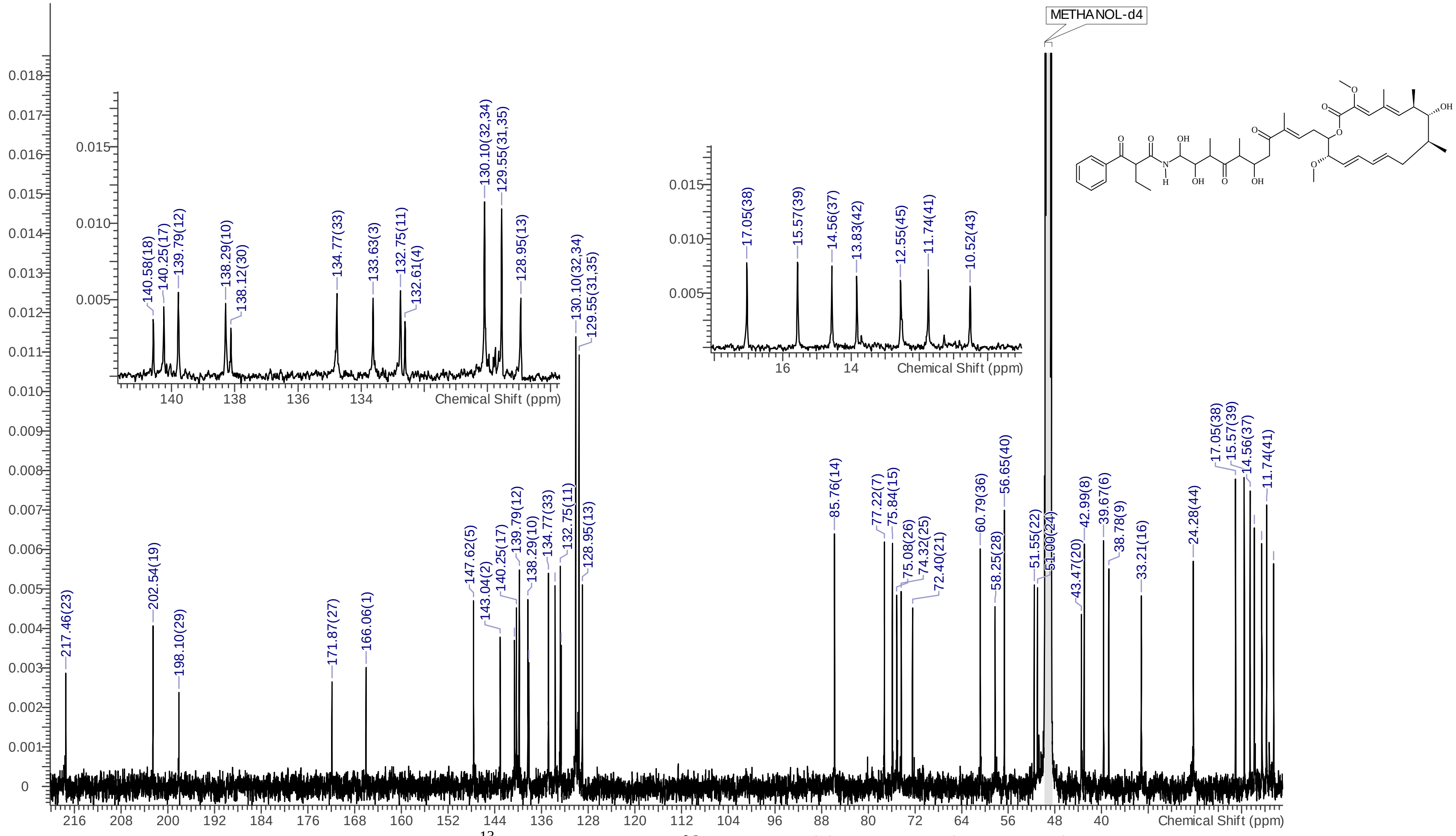

Figure S2. ^{13}C NMR spectrum of lanyamycin (**1**) in CD_3OD (125.8 MHz)

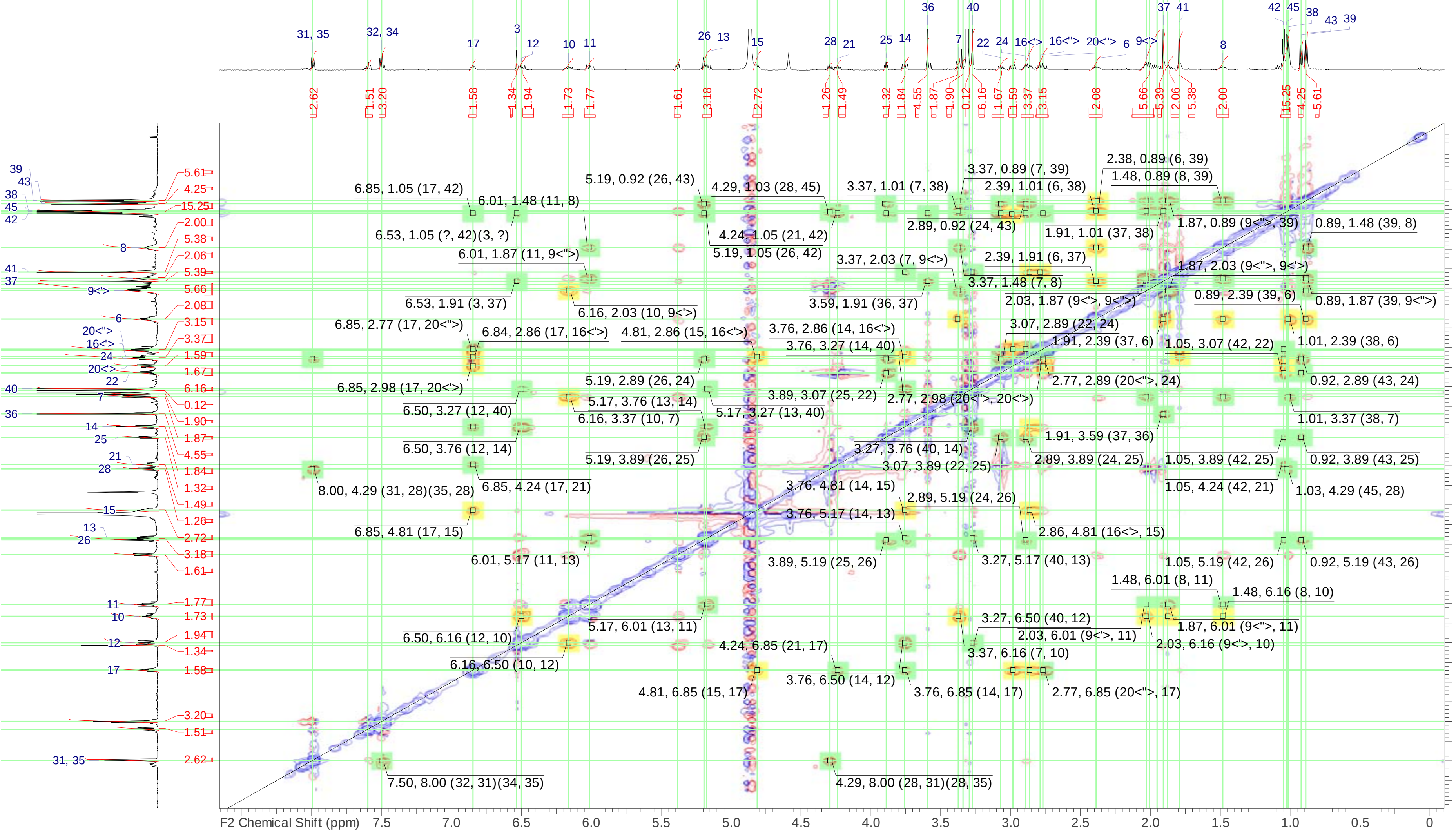

Figure S3. ROESY NMR spectrum of lanyamycin (**1**) (1H/13C/15N NMR at 500.3/125.8/50.7 MHz)

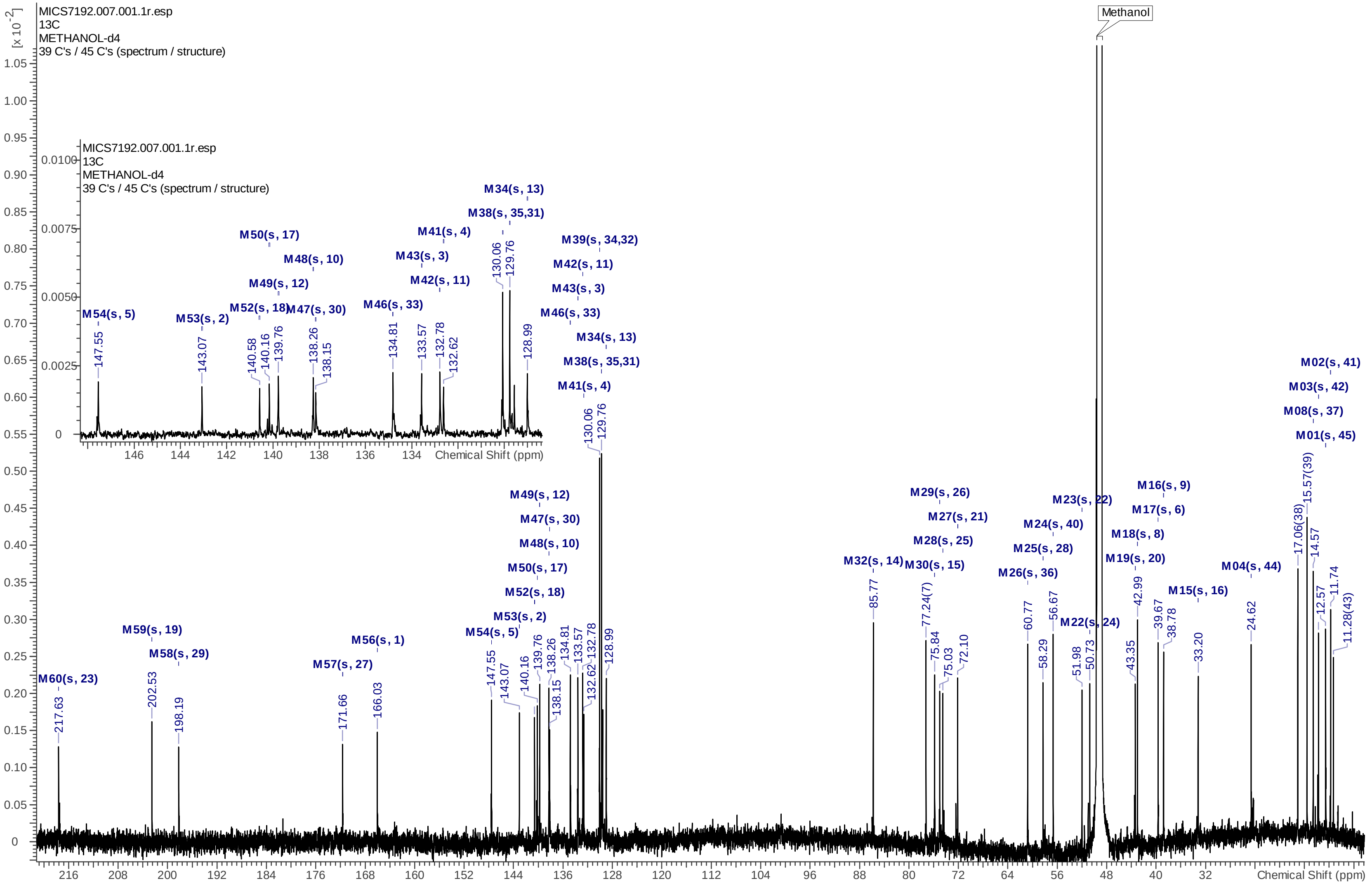

Figure S4. ^{13}C NMR spectrum of lanyamycin (**2**) in CD_3OD (125.8 MHz)

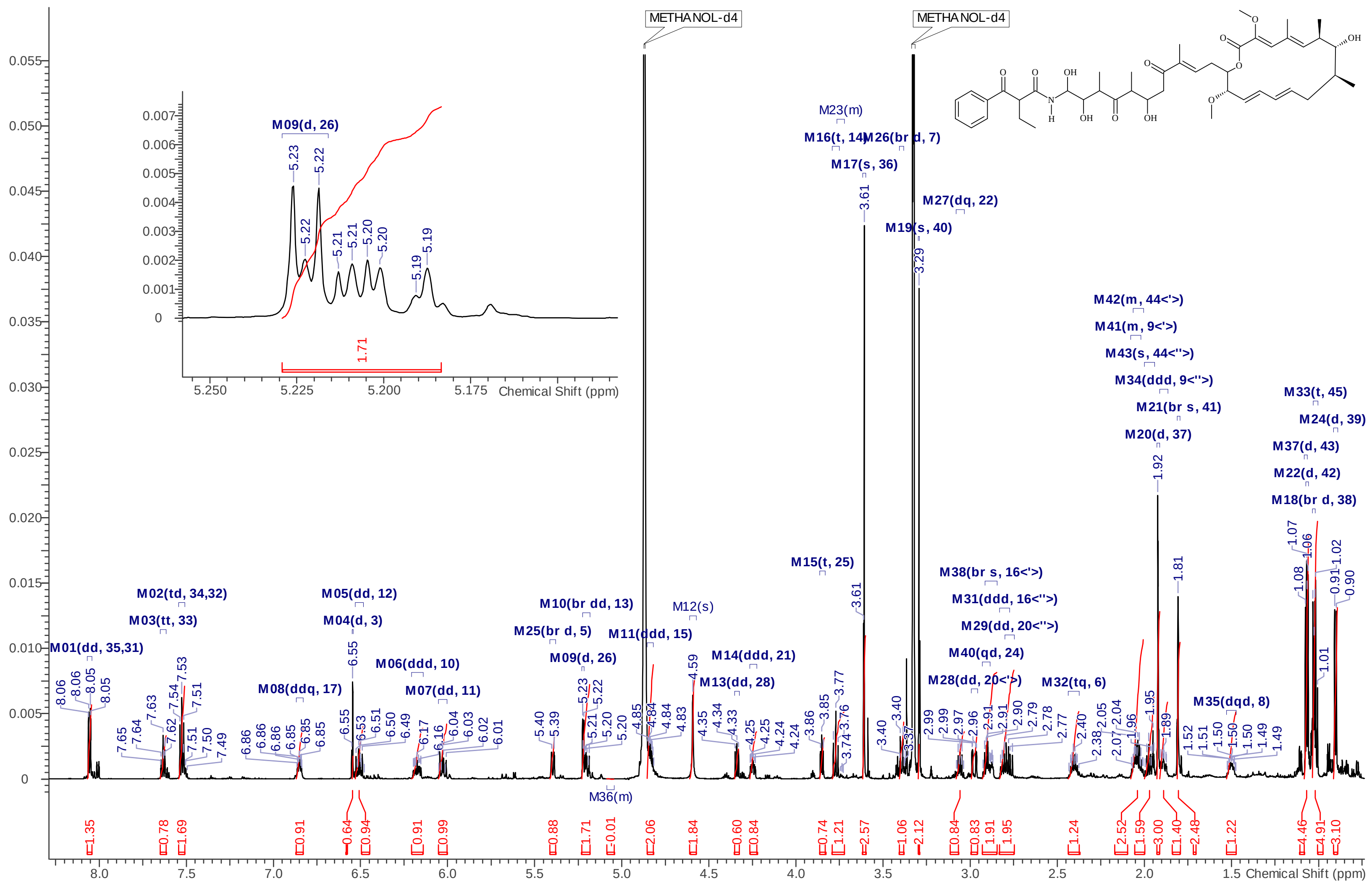

Figure S5. ^{1}H NMR spectrum of lanyamycin (**2**) in CD_3OD (500.3 MHz)

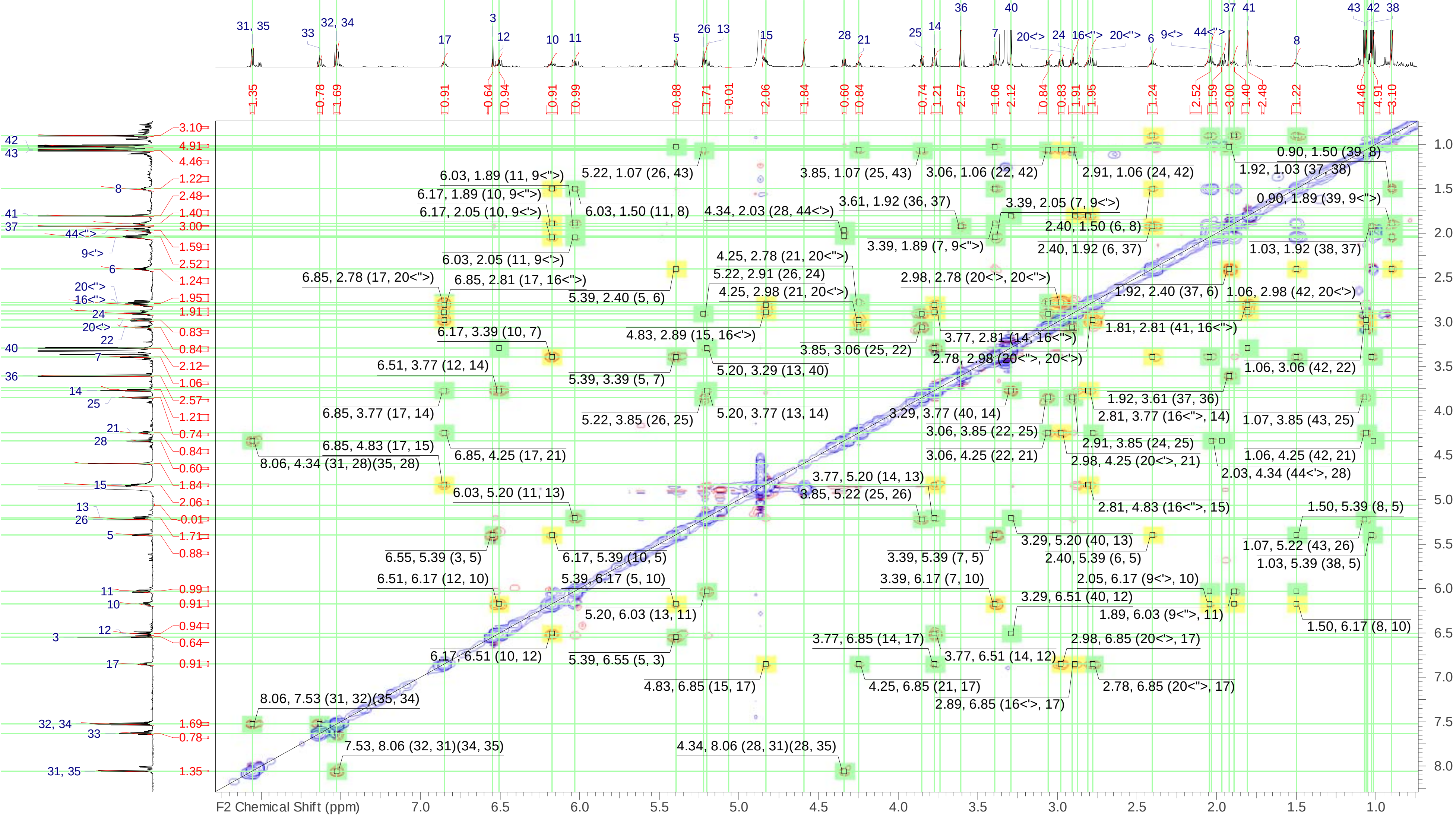

Figure S6. ROESY NMR spectrum of lanyamycin (**2**)

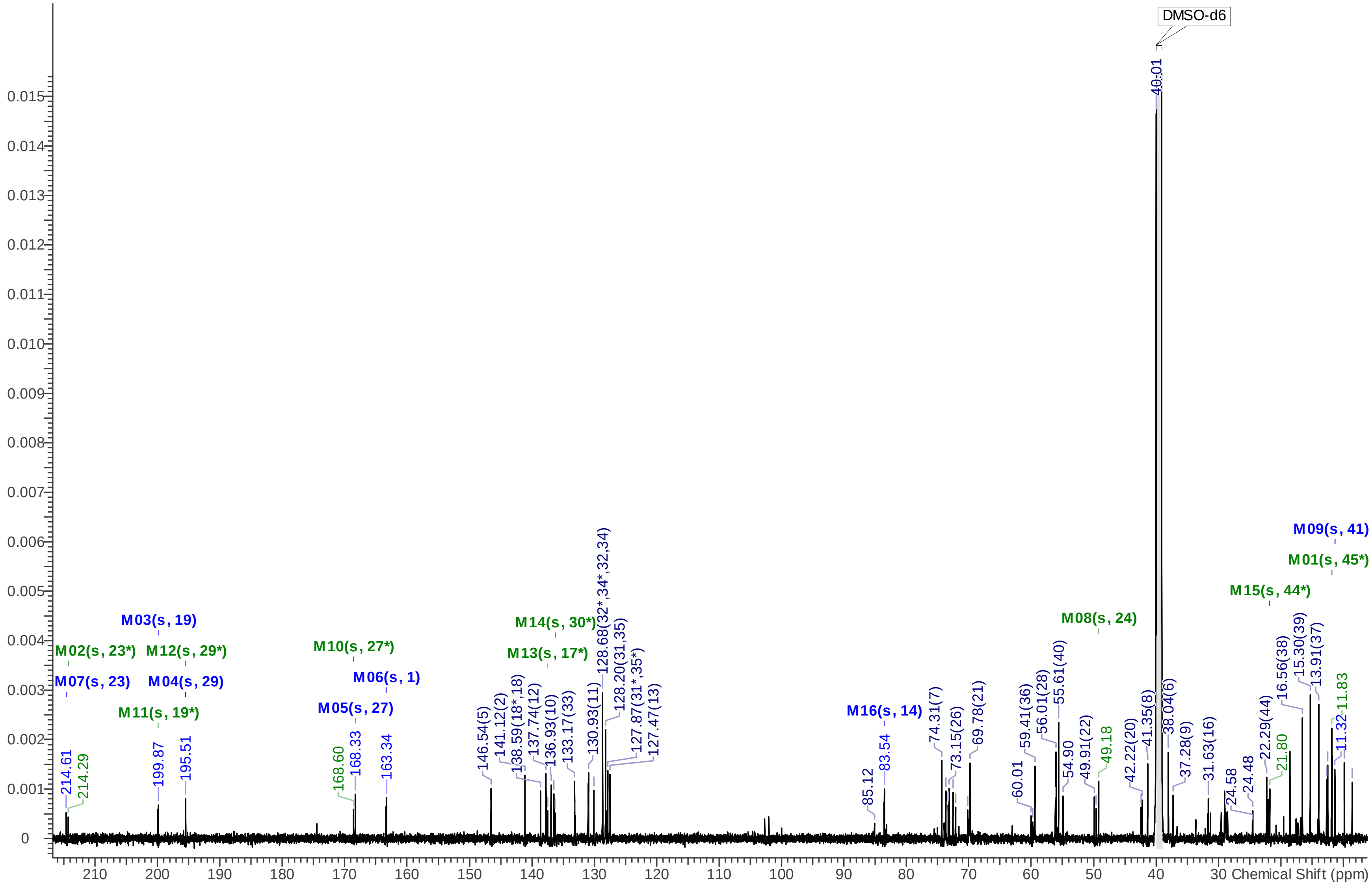

Figure S7. ^{13}C NMR spectrum of lanyamycin (**1**/**2**) in DMSO-d6 (125.8 MHz)

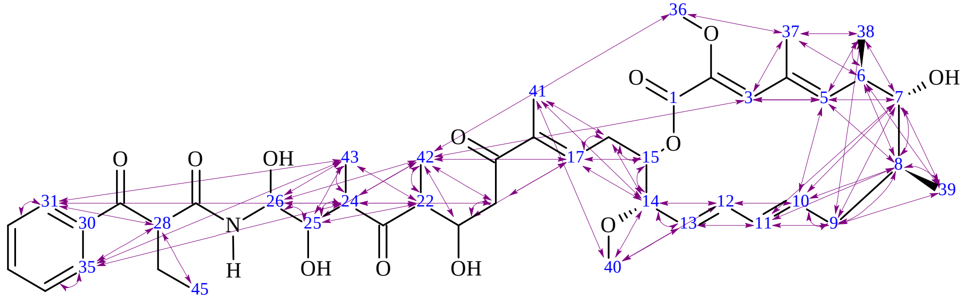

Lanyamycin **1**

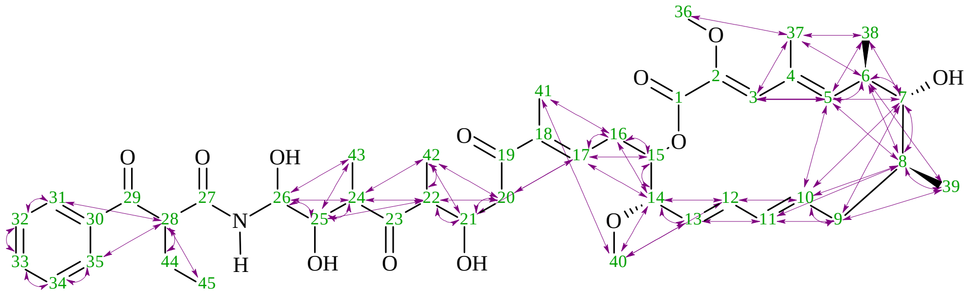

Lanyamycin **2**

Figure S8. Comparison of the NOE intensities of lanyamycin **1** and **2**

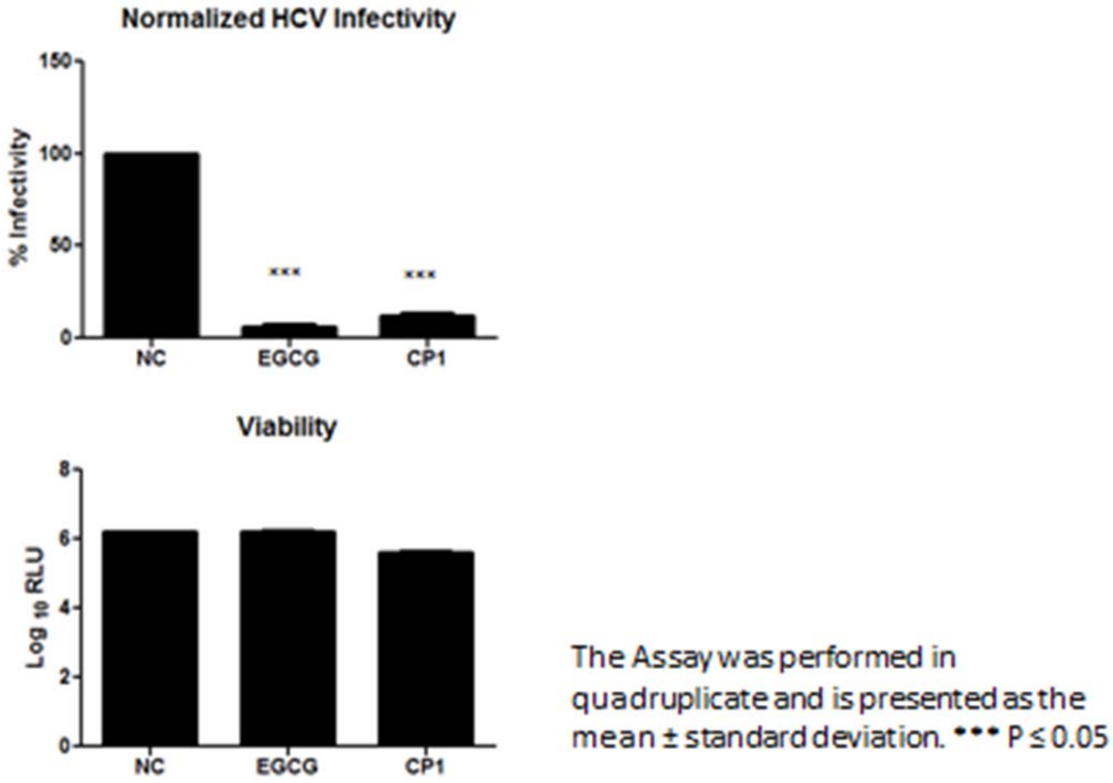

Figure S9. Lanyamycin (**1**/**2**) effects on HCV infectivity including viability assay

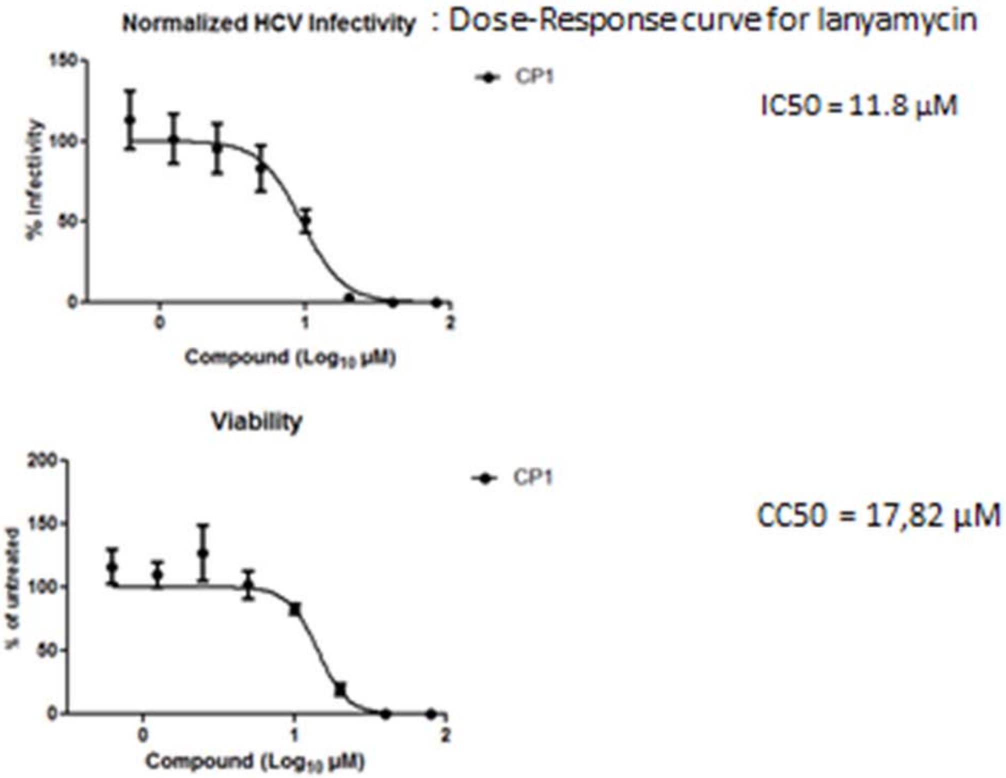

Figure S10. Dose-Response curve for lanyamycin (**1**/**2**) HCV infectivity
